# RESILIENCY AMONG WHI’S WOMEN AGED 80 AND OLDER BY RACE, ETHNICITY, AND NEIGHBORHOOD SOCIOECONOMIC STATUS

**DOI:** 10.1093/geroni/igae098.1351

**Published:** 2024-12-31

**Authors:** Jessica Krok-Schoen

**Affiliations:** The Ohio State University, Columbus, Ohio, United States

## Abstract

A comprehensive examination of resilience by race, ethnicity, and neighborhood socioeconomic status (NSES) among women aged ≥80 is needed, given the aging U.S. population, increasing longevity, and growing racial and ethnic diversity. Participants (n=29,367, median age=84.3) were women aged ≥80 enrolled in the Women’s Health Initiative. There were no significant differences by race and ethnicity in resiliency. Mean resiliency scores differed between those with low NSES (3.94±0.83) and high NSES (4.00±0.81). Older age, higher education, higher self-rated health, lower stress, and living alone were correlates of resilience. Social support correlated with resilience among White, Black, and Asian women, but not for Hispanic women. Living alone, smoking, and spirituality were significantly associated with higher resilience among women with moderate NSES. Despite some differing correlates of resilience by race, ethnicity, and NSES, there were many similarities. These results may aid in designing resilience interventions for the increasingly diverse population of older women.

